# Differential Response of Acidobacteria to Water Content, Soil Type, and Land Use During an Extended Drought in African Savannah Soils

**DOI:** 10.3389/fmicb.2022.750456

**Published:** 2022-02-11

**Authors:** Katharina J. Huber, Selma Vieira, Johannes Sikorski, Pia K. Wüst, Bärbel U. Fösel, Alexander Gröngröft, Jörg Overmann

**Affiliations:** ^1^Leibniz Institute DSMZ—German Collection of Microorganisms and Cell Cultures, Braunschweig, Germany; ^2^Department of Geosciences, Institute of Soil Science, University of Hamburg, Hamburg, Germany; ^3^Institute of Microbiology, Technical University Braunschweig, Braunschweig, Germany

**Keywords:** acidobacteria, drought, subtropical savannah soils, environmental parameters, high-throughput sequencing

## Abstract

Although climate change is expected to increase the extent of drylands worldwide, the effect of drought on the soil microbiome is still insufficiently understood as for dominant but little characterized phyla like the Acidobacteria. In the present study the active acidobacterial communities of Namibian soils differing in type, physicochemical parameters, and land use were characterized by high-throughput sequencing. Water content, pH, major ions and nutrients were distinct for sandy soils, woodlands or dry agriculture on loamy sands. Soils were repeatedly sampled over a 2-year time period and covered consecutively a strong rainy, a dry, a normal rainy and a weak rainy season. The increasing drought had differential effects on different soils. Linear modeling of the soil water content across all sampling locations and sampling dates revealed that the accumulated precipitation of the preceding season had only a weak, but statistically significant effect, whereas woodland and irrigation exerted a strong positive effect on water content. The decrease in soil water content was accompanied by a pronounced decrease in the fraction of active Acidobacteria (7.9–0.7%) while overall bacterial community size/cell counts remained constant. Notably, the strongest decline in the relative fraction of Acidobacteria was observed after the first cycle of rainy and dry season, rather than after the weakest rainy season at the end of the observation period. Over the 2-year period, also the β-diversity of soil Acidobacteria changed. During the first year this change in composition was related to soil type (loamy sand) and land use (woodland) as explanatory variables. A total of 188 different acidobacterial sequence variants affiliated with the “*Acidobacteriia*,” *Blastocatellia*, and *Vicinamibacteria* changed significantly in abundance, suggesting either drought sensitivity or formation of dormant cell forms. Comparative physiological testing of 15 Namibian isolates revealed species-specific and differential responses in viability during long-term continuous desiccation or drying-rewetting cycles. These different responses were not determined by phylogenetic affiliation and provide a first explanation for the effect of drought on soil Acidobacteria. In conclusion, the response of acidobacterial communities to water availability is non-linear, most likely caused by the different physiological adaptations of the different taxa present.

## Introduction

Due to climate change, the incidence and length of drought events increases worldwide (Touma et al., 2015).^1^ Particularly in permanent or seasonally arid drylands which cover ∼41% of the land masses (Maestre et al., 2015), extended drought can result in crop failure and famine of the local human population. Although the soil microbiome mediates relevant ecosystem functions like nitrogen fixation, decomposition of detritus and a suite of different (exo) enzymatic activities (Kundu and Gaur, 1980; Baumann et al., 2013) which are of direct relevance to soil fertility, the effects of drought on soil bacteria is to date only little understood (Maestre et al., 2015; Hartmann et al., 2017; Naylor and Coleman-Derr, 2018).

Numerous representative species of the three bacterial phyla Proteobacteria, Firmicutes, and Actinobacteria that are highly abundant in soil have been isolated and described (Overmann et al., 2017). Some of these species are able to survive drought through the production of exospores, myxospores, or endospores (Beskrovnaya et al., 2021).

However, drought survival strategies have not been determined for the majority of the soil bacterial microbiome, including another dominant group, the Acidobacteria.

Acidobacteria, despite representing 5–70% of the soil bacterial communities (Janssen, 2006; Jones et al., 2009; Lauber et al., 2009), have remained largely understudied. As a consequence, the environmental drivers of the acidobacterial community composition and the specific niche adaptations of the phylogenetically highly diverse acidobacterial taxa are mostly unknown. So far, analysis of the distributional patterns of acidobacterial 16S rRNA sequences identified pH as a major environmental determinant. Thus, members of the classes *Blastocatellia* and *Vicinamibacteria* dominate soils with a neutral or slightly basic pH, whereas “*Acidobacteriia*” (*Acidobacteriales* and *Bryobacterales*) prevail in soils with more acidic pH values (Jones et al., 2009; Naether et al., 2012; Foesel et al., 2014; Ivanova et al., 2020). So far, either an overall decrease in the abundance of soil or root-associated Acidobacteria during drought (Barnard et al., 2013; Maestre et al., 2015; Santos-Medellín et al., 2017), or an enrichment in desiccated roots or soils have been documented (Desgarennes et al., 2014; Yuste et al., 2014; Naylor and Coleman-Derr, 2018). Acosta-Martínez et al. (2014) described Acidobacteria as drought sensitive, whereas Hartmann et al. (2017) proved that the abundance of several acidobacterial groups increased with long-term water limitation in semi-arid pine forest soils while Acidobacteria.6 (∼*Vicinamibacteria*) decreased with drought. To date, 22% of all known and validly named acidobacterial species originate from Namibian subtropical savannah soils (Foesel et al., 2013, 2015; Huber et al., 2014, 2016; Pascual et al., 2015a,b; Wüst et al., 2016). Since this region is subject to recurring dry seasons, its soils and the corresponding acidobacterial isolates provide the opportunity to study the effect of changing water availabilities on different acidobacterial taxa in more detail.

In order to examine the influence of drought events on the acidobacterial community and the response mechanisms of Acidobacteria toward varying soil water availability, acidobacterial communities were tracked by Illumina high-throughput sequencing in 96 subtropical savannah soils of the Kavango region in North-Eastern Namibia. The soils differed in soil type, physicochemical characteristics and land use, and were sampled over two cycles of dry and rainy seasons and increasing drought. Complementing the culture-independent data, 15 isolates (representing 14 novel species) of Acidobacteria that are currently available from Namibian soils were characterized with respect to their adaptation to continuous desiccation and drying-rewetting cycles. Our results offer novel insights into the differential adaptation of various acidobacterial taxa to drought events in semi-arid soils.

## Materials and Methods

### Study Sites

**FIGURE 1:**
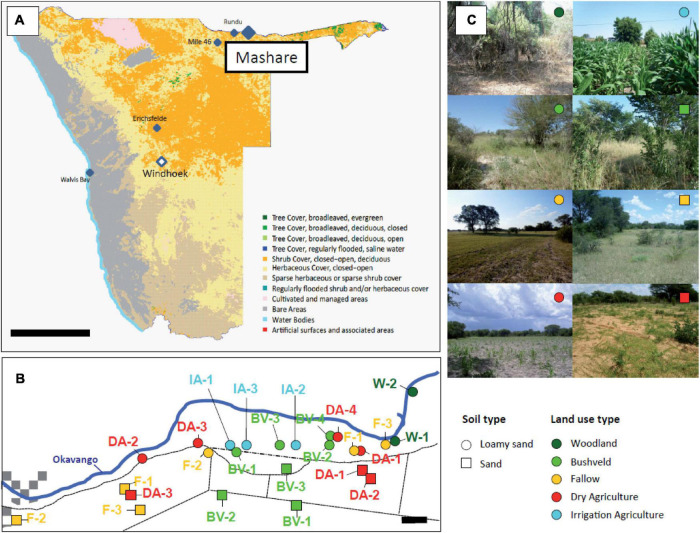
**(A)** Overview map of Namibia with the highlighted sampling areas in Mashare, Rundu (N’Kwazi Lodge) and Erichsfelde. Bar, 300 km. **(B)** Detailed map of the sampling locations in the Mashare region. Bar, 1 km. **(C)** Representative photographs of the eight different types of sampling locations present in Mashare. In **(B,C)** soil types (loamy sand, circles; and sand, squares) and land use types (woodland, dark green; bushveld, green; fallow, yellow; dry agricultue, red; irrigation agriculture, blue) are denoted by different symbols.

The 96 soil samples analyzed in this study were collected in Mashare, North-East Namibia (17°53′40.9′′S, 20°10′39.6′′E; 1,070 m above sea level) as a part of the BMBF funded project “The Future Okavango” (Figure 1A and Supplementary Table 1). Within the four different sampling campaigns, two to three biological replicates were chosen for each existing combination of soil type (sand or loamy sand) and land use type (woodland, bushveld, fallow, dry agriculture, irrigation agriculture) (Figures 1B,C). The soils chosen were representative for the old river terraces of the Okavango river (loamy sands) as well as the adjacent subfossil Kalahari dunes (sands).

**FIGURE 2:**
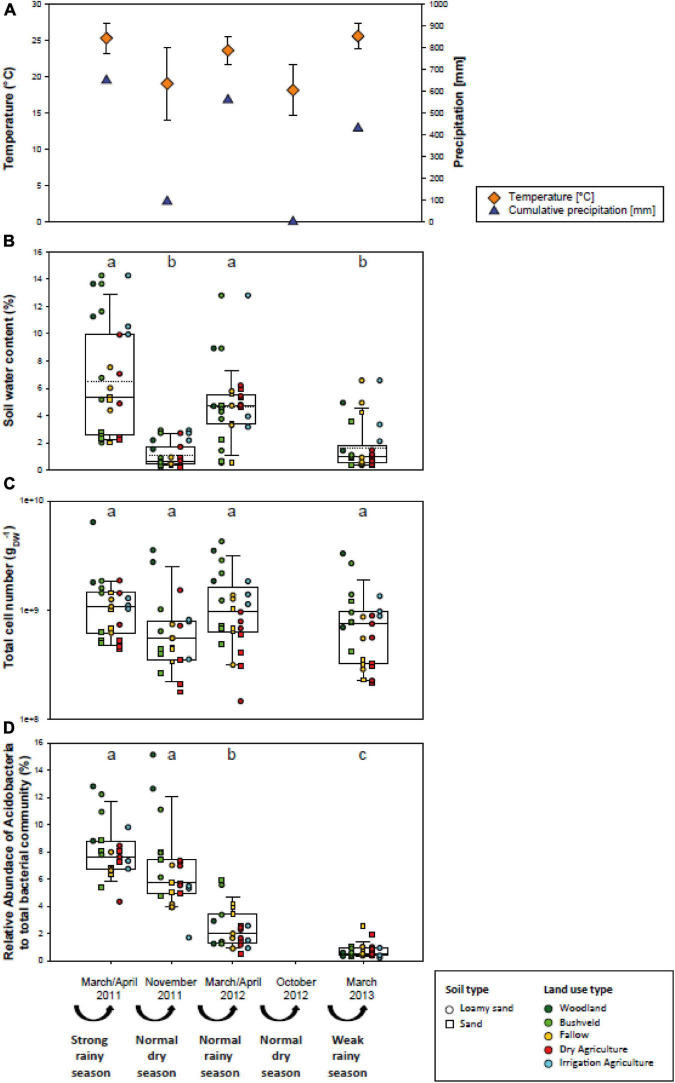
Environmental parameters in different Mashare soils in March/April 2011 after a strong rainy season, in November 2011 after a dry season, in March/April 2012 after a normal rainy season, and in March 2012 after a weak rainy season. **(A)** Mean temperature values in°C (*n* = 30; orange diamonds; means and standard deviations are given) and cumulative precipitation values of the preceding season in mm (blue triangles). **(B)** Soil water content (%). **(C)** Total cell numbers (cells g_*DW*_^– 1^). **(D)** Relative abundance values of Acidobacteria among the total bacterial community determined in the Mashare soils collected at four time points (%). Values for individual sampling plots are given and denoted according to their soil and land use types (compare Figure 1). Lower and upper hinges of boxplots correspond to the first and third quartiles. Upper whisker extends from the hinge to the largest value no further than the 1.5 inter-quartile range and lower whisker from the hinge to the smallest value with at most 1.5 times the inter-quartile range. Different letters in lower case stand for statistically significant differences at *p* < 0.01.

The Mashare region features a hot semiarid climate with dry winters. Rainfall occurs during the summer months between November and March and is followed by a dry season from April to October. The annual average precipitation in Mashare is 595 mm and the annual mean temperature is 22.3°C (AQUASTAT, 2014). However, the four sampling campaigns in Mashare were characterized by considerable variations in precipitation. In the rainy season of March/April 2011 the heaviest rainfalls occurred in Namibia since 120 years (649 mm) followed by a dry season with rainfalls in April and May which lasted until November 2011 (92 mm). Until March/April 2012 normal rain falls were monitored (559 mm), followed by 0 mm until November 2012 and further decreased rainfalls in the rainy season of 2013 (429 mm) (Figure 2A).

### Sample Collection

Between 23 and 25 samples each were collected in March/April 2011, November 2011, March/April 2012, and March 2013 (Supplementary Table 1). After removing the litter layer, the upper 10 cm of the soils were collected along two perpendicular transects that covered 5 sampling spots each, spaced 5 m apart. All subsamples were pooled and homogenized. One aliquot of the pooled soil sample was kept at 4°C during the transport to the laboratory and served as inoculum for the cultivation of Acidobacteria. For the determination of the composition of the soil microbial community another soil aliquot was either directly frozen in liquid nitrogen or treated with LifeGuard™ Soil Preservation Solution (MoBio Laboratories, Carlsbad, CA, United States) with subsequent freezing at –80°C until extraction of the nucleic acids. Due to logistical reasons the samples of the campaigns March/April 2011 and November 2011 were frozen in liquid nitrogen, whereas the samples of the campaigns March/April 2012 and March 2013 had to be treated with LifeGuard™ Soil Preservation Solution directly in the field. A direct comparison of several additional samples taken in March/April 2011 and treated with LifeGuard™ Soil Preservation Solution did not yield any distinct differences in the acidobacterial community composition (data not shown). Additionally, larger samples obtained in March/April 2011 and 2012 were maintained at room temperature for subsequent physicochemical soil analyses.

### Physicochemical Soil Parameters and Total Cell Counts

Different soil parameters have been determined to identify the potential environmental key drivers of the soil microbiome. Soil temperature was determined directly in the field in the top 10 cm of the soils (Checktemp 1 thermometer; Hanna Instruments, Kehl). Dry combustion analysis (Vario Max; Elementar Analysensysteme GmbH, Hanau, Germany) was employed to determine total carbon and total nitrogen contents. If present, inorganic carbon was measured on a dry fine-ground sample treated with 5 ml phosphoric acid (43%) in a closed system and measuring the released CO_2_ by gas chromatography. Organic carbon was calculated as the difference between total and inorganic carbon. Phosphorus concentrations were quantified in a double-lactate extract and the amounts of K^+^, Na^+^, Mg^2+^, Ca^2+^, Fe^2+/3+^, Cl^–^, NO_3_^–^, NO_2_^–^, SO_4_^2–^, and HCO_3_^–^ in aqueous extracts of the soils [1:1 (w/v)], respectively (Herpel, 2008). The soil water content was determined after drying at 80°C for 72 h. The pH values were measured in distilled water as well as in 10 mM CaCl_2_ solution.

Total cell numbers in the soil samples were determined by SYBR^®^ Green I staining according to the method described by Lunau et al. (2005). 0.1 g of soil were fixed with 900 μL MES buffer (pH 6.0, 10 mM) containing 1% (v/v) glutaraldehyde (final concentration). Then 50 μL of the suspension were mixed with methanol and MES buffer (pH 5.5, 10 mM) and treated in the ultrasonic bath for 15 min at 35°C. 500 μL of the suspension was added to 9.5 ml MOPS buffer (2 mM, pH 7.0) and stained with 2 μL SYBR^®^ Green I staining solution. After 10 min staining in the dark the cells were collected on black polycarbonate filters (Millipore GTBP 0.2 μm), covered with DABCO and counted in a fluorescence microscope (Zeiss Imager M2, GFP Filter, Jena, Germany).

### RNA-Extraction

To identify the active fraction of the soil microbiome and thereby its directs response, RNA was extracted, transcribed in cDNA for amplicon preparation, subsequently sequenced and bioinformatically analyzed.

RNA was extracted from soil samples using the method described in Lueders et al. (2004). A soil aliquot of 0.6 g (fresh weight) was subjected to beat beating (FastPrep^®^-24 Instrument; MP Biomedicals, Carlsbad, CA, United States) in sodium phosphate buffer (120 mM) containing sodium dodecyl sulfate (30%; w/v). Samples were centrifuged for 20 min at 20817 × g (Eppendorf 5417R, Hamburg, Germany) and the aqueous supernatant was extracted with one volume of phenol/chloroform/isoamylalcohol (25:24:1; v/v/v) and one volume of chloroform/isoamylalcohol (24:1, v/v). For precipitation of the nucleic acids, two volumes of polyethylene glycol were added and the extracts were centrifuged for 90 min at 4°C and 20817 × g. The nucleic acid pellets were washed with ice cold 70% (v/v) EtOH and resuspended in 50 μL elution buffer (10 mM Tris-HCl, pH 8.5).

Subsequently, the DNA in the extracts was digested with RNase-free DNase I (1 U/μL; Fermentas, Thermo Fisher Scientific, Waltham, MA, United States) according to the instructions of the manufacturer. The RNA was precipitated with RNase free sodium acetate (pH 5.2) and isopropanol. After incubation on ice and centrifugation, the RNA pellet was washed once with EtOH (70% v/v) and resuspended in 30 μL pure PCR water (Sigma, St. Louis, MO, United States).

### Preparation of V3-Amplicons and Illumina HighSeq Sequencing

The GoScript™ Reverse Transcriptase System (Promega, Madison, WI, United States) was employed according to the instructions of the manufacturer to synthesize cDNA from the RNA. Amplicons of the V3-region of the 16S rRNA were prepared following the method of Bartram et al. (2011) that allowed multiplexing of multiple samples for one high-throughput sequencing run. The amplification of the V3 region of the 16S rRNA gene was performed in the Veriti^®^ Thermal Cycler employing the Phusion^®^ High-Fidelity DNA Polymerase Kit (New England Biolabs, Ipswich, MA, United States), the modified forward primer V3_F (50 pmol/μL; aatgatacggcgaccaccgagatctacactctttccctacacgacgct cttccgatctCCTACGGGWGGCWGCAG) and the modified reverse primer V3_xR (50 pmol/μL; caagcagaagacggcatacgaga tNNNNNNgtgactggagttcagacgtgtgctcttccgatctCCGCGGCTGCT GGCAC). The reaction mix was incubated for 5 min at 94°C for initial denaturation of the DNA. 15 cycles of denaturation (15 s at 94°C), elongation (15 s at 59°C) and annealing (15 s at 72°C) followed and the amplification was finished by a terminal annealing step for 7 min at 72°C.

Primer dimers were removed by gel purification using MetaPhor^®^ agarose (Lonza, Basel, Switzerland). Afterward the purified DNA was extracted using the NucleoSpin^®^ Gel and PCR Clean-up Kit (Macherey-Nagel, Merck, Darmstadt, Germany). Finally, the cDNA amplicons deriving were sequenced on an Illumina Genome Analyzer and an Illumina HiSeq 2500 Ultra-High-Throughput Sequencing System (Illumina, San Diego, CA, United States). On average a total of 2–3 million reads per sample were generated during one 100 paired-end run.

### Bioinformatic Analysis

The generated V3 amplicon sequences were run through a bioinformatic analysis pipeline which allows the processing of multimillions of amplicon reads and consists of the following steps. Raw sequence reads were processed in QIIME 2™ (Version 2019.10^2^; Bolyen et al., 2019). Raw forward and reverse sequence reads were imported, joined and quality filtered using the “vsearch,” “quality-filter,” and “deblur” (trim-length of 165 bp) plugins with default options (Bokulich et al., 2013; Amir et al., 2017). Representative sequences were aligned to reconstruct a midpoint-rooted tree using the “alignment” and “phylogeny” plugins. Taxonomic classification of representative sequences was done using a Naïve Bayes classifier which was trained on the V3 region of the 16S rRNA gene sequence database of SILVA (version 132) (Quast et al., 2013) using the plugin “feature-classifier.” For further statistical analysis the read count table (table.qza), the taxonomic classification (taxonomy.qza) and the phylogenetic tree (rooted-tree.qza) were exported and then imported into the R environment, version 3.2.3 (R Core Team, 2020). All Illumina datasets were submitted to the ENA database under the study accession number PRJEB46595.

### Multivariate Statistical Analyses

All statistical analyses were done in R version 4.1.0. Statistically significant differences at *p* < 0.01 in pairwise group means of multiple group comparisons (Tukey procedures) were tested with the functions glht (aov(), vcov = vcovHC) in “multcomp” version 1.4 (Herberich et al., 2010) and utilizing a heteroskedasticity-consistent covariance matrix estimation using function vcovHC() from “sandwich” version 2.4 (Zeileis, 2004). Multivariate ordinations (PCA, PCoA) and Procrustes analyses were performed using the vegan R package version 2.5–7 (Oksanen et al., 2018). PERMANOVA analyses were performed using the function adonis2() in the vegan package and were repeated 100 times in order to account for stochastic variability during the permutation procedure (*N* = 999 by default) and to determine the mean and standard deviation of the *p*-value. Physicochemical soil variables were scaled [rda(Input_Data, scale = TRUE)] for the Principal Component Analysis. Soil water content as response variable was analyzed by linear modeling (function lm in R) across the sampling time points November 2011, March/April 2012 and March 2013. Soil water content of the prior sampling campaign and the cumulative precipitation values prior to the current sampling campaign were used as fixed numerical explanatory variables, whereas soil type and land use type were employed as fixed categorical explanatory variables. The cumulative precipitation values were divided by 100 to adjust the scale to that of the soil water content values. Gradient boosting with stability selection was used for variable selection and performed with the mboost R package version 2.9–5 (Hothorn et al., 2020) and the R package stabs version 0.6–4 (Hofner et al., 2015). Gradient boosting is a machine learning algorithm which is used to optimize the estimation of classical statistical models (Mayr and Hofner, 2018). Stability selection allows to apply gradient boosting to data situations in which there are much less observations per explanatory variable than typically suggested for statistical models (≥10). The categorical levels for soil type and land use were entered as dummy variables (0 for absent, 1 for present). The initial gradient boosting model was obtained with the parameters INIT_model < – glmboost (Input_Data, control = boost_control (mstop = 1,000). Visual inspection of INIT_model ensured that all explanatory variables were captured. Stability selection was performed with the parameters stabsel (INIT_model, cutoff = 0.8, *q* = 2, sampling.type = “MB”) in order to keep the false-positive detection rate low. Each stability selection run was performed 50 times in order to account for stochastic variations in the runs. Only selection probabilities > 0.5 are regarded to be stable enough to be taken into account as explanatory variable.

### Differential Abundance Testing

To identify sequence variants (SVs) which were influenced by the increasing drought, the differential relative abundances (fold changes) between all March/April sampling campaigns were determined in a pairwise manner. SVs which differed significantly in relative abundances were identified using the ANCOMBC R package (Lin and Peddada, 2020) at *p* < 0.05 (Benjamini-Hochberg corrected *p*-values). The log2 fold changes were calculated with the DESeq2 R package.

### Phylogenetic Clustering of Drought-Sensitive Sequence Variants

The phylogenetic clustering of SVs enriched between sampling dates was analyzed by calculating the net relatedness index (NRI) and nearest taxon index (NTI) (Webb et al., 2002) values using the functions ses.mpd() and ses.mnt() of the *picante* R package, respectively. Positive values show that SVs are more related to each other than what is predicted by random models. While NRI indicates phylogenetic clustering at deep branches, positive NTI values reveal a predominant clustering at the terminal branches. NRI and NTI increase with increasing clustering of lineages with the same trait and become negative with overdispersion. Maximum conservatism in traits at a deep branching level yields high values of NRI as well as NTI. Conservatism at terminal branches of the phylogeny result in NTI that increase in significance relative to NRI (Webb et al., 2002). For these calculations, only SVs which were present in a minimum of two samples were considered.

### Phylogenetic Tree of Namibian Isolates

In the past years a total of 16 strains were isolated from Namibian soil samples of Erichsfelde (*n* = 4), Mashare (*n* = 10) and Rundu (*n* = 2) (Figure 1A and Supplementary Table 2) by high-throughput-cultivation methods. All strains were physiologically characterized and the data already mainly published (Foesel et al., 2013, 2015; Huber et al., 2014, 2016; Pascual et al., 2015a,b; Wüst et al., 2016).

A phylogenetic tree was calculated with the maximum-likelihood method for all known acidobacterial strains with (validly) pulished names to show their affiliation with the higher taxonomic ranks. The ARB software (Ludwig et al., 2004) and the SILVA database 132 (Pruesse et al., 2007) were employed to run a 1000 bootstrap tree to show the phylogenetic affiliation of the Namibian isolates within the Acidobacteria. The presence of the sequences of the acidobacterial isolates within the amplicon datasets from the different Mashare soils was assessed using BLAST at a similarity level of 100%.

### Effects of Drought on the Viability of Different Acidobacterial Isolates

The effect of decreasing water availability on the viability of 15 Namibian isolates (Supplementary Table 2) was tested in an artificial soil matrix (Winterstein, 2003) composed of 82.00 g sand (sea sand, grain size 100–300 μm, Merck, Darmstadt, Germany), 2.88 g kaolinite (Merck), 4.86 g montmorillonite (Sui Jin, Japan) and 10.26 g illite (Argiletz, Lizy sur Ourcq, France) per 100 g. The artificial soil matrix was sterilized by autoclaving.

Strains were grown in liquid SSE/HD 1:10 medium (soil solution equivalent/yeast dextrose 1:10 medium; DSMZ medium 1426^3^; pH 5.5 or 7.0 according the growth optimum of the strains) until the late-exponential phase as determined by optical density measurement (Thermo Fisher Scientific, Genesys 20, Waltham, MA, United States). Cells were harvested by centrifugation (10,000 × g, Beckman Coulter Avanti^®^ J-30I/J-26 XPI, Galway, Ireland) for 15 min and room temperature and then washed either with MES (10 mM, pH 5.5) or HEPES buffer (10 mM, pH 7.0) depending on the pH optimum of the respective isolates. Finally, cells were resuspended in 1x SSE (soil solution equivalent, buffered with MES or HEPES). For each strain, 5 ml of 1x SSE containing approximately 10^9^ cells were spread onto and mixed with 20 g of the artificial soil matrix and incubated. This treatment resulted in an initial soil water content of 20%.

For the simulation of drought, two parallel approaches were used. In one set of experiments, the inoculated artificial soil matrix was incubated at 28°C without any further treatment, resulting in a continuous decline of soil water content over the incubation period. Latest at time point 70 d a soil water content of 0% was determined. In a parallel set of experiments, the inoculated artificial soil matrix was subjected to repeated drying/rewetting cycles. For this purpose, the artificial soil was dried for 5 h at 50°C and then incubated in an exsiccator (Glaswerk Wertheim, Germany) with silica gel overnight. The next day the water content was readjusted with sterilized ddH_2_O (filtered with 0.1 μm filter, Millipore, Billerica, MA, United States) to a value of 20% and the soil subsequently incubated at 28°C. Additional drying-rewetting cycles were performed after incubations at 7, 14, 21, 28, and 35 d. After the fifth drying-rewetting cycle, the soil was finally kept at 28°C.

For the determination of the colony forming units, 0.1 g of the soil of the two parallel sets of experiments were harvested at the time points 0, 1, 7, 8, 14, 15, 21, 22, 28, 29, 35, 36, 70, or 77 d and 100 or 108 d (i.e., intermittently and directly after drying rewetting during the respective experiments). The soil was mixed with MES or HEPES and serially diluted. 100 μL of the soil slurries were plated in triplicates on SSE/HD 1:10 medium. Since the doubling time of the isolates has been described to be rather low (4.4–17.4) (Foesel et al., 2013, 2015; Huber et al., 2014, 2016; Pascual et al., 2015a,b; Wüst et al., 2016), numbers of colonies were counted after 2 months of incubation to determine the viability values.

## Results

### Changes in Soil Water Content and Its Environmental Determinants

The rainy season of 2010/2011 in Namibia was the strongest rainy season observed during the past 120 years and was followed by a normal rainy season in 2011/2012 and a weak rainy season in 2012/2013. In the Mashare region, cumulative precipitation over the whole rainy season of 2010/2011 amounted to 649 mm, in the rainy season of 2011/2012 to 559 mm, and in the rainy season of 2012/2013 to 429 mm (Figure 2A). Whereas cumulative precipitation in the intermittent dry season of 2011 was 93 mm, no precipitation was recorded in the following dry season of the year 2012. Over the 2-year study period, the mean air temperature during rainy seasons varied between 23.6 and 25.6°C, and the two dry seasons reached values of 19.0 and 18.0°C (Figure 2A).

**FIGURE 3:**
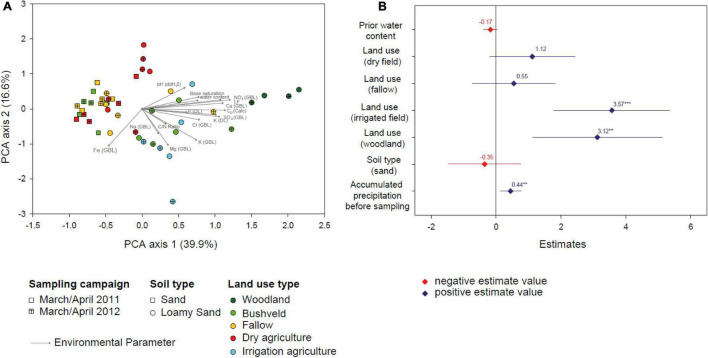
**(A)** Biplot of a Principal Component Analysis of 14 physicochemical soil variables (specified in the text) for soil samples taken in March/April 2011 and 2012. Symbols give scores for sampling sites and arrows point to the scores of the soil variables. Shape of the symbols indicates soil type and time point of sampling, color indicates land use types. **(B)** Estimates of a linear model with soil water content as response variable. Diamonds represent values for the estimates and lines represent the 95% confidence intervals. Stars denote estimates that differ significantly from zero (**p < 0.01; ***p < 0.001).

Over the sampling period, soil water content varied considerably. Similarly to the cumulative precipitation of the preceding season, median soil water content exhibited highest values in the rainy season of 2010/2011 (5.4%) and decreased in the rainy season 2011/2012 (4.7%). Lowest values were measured in the dry season of 2011 and the rainy season in 2012/2013 (0.66 and 0.96%, respectively) (Figure 2B). The examined soils differed in type (sand or loamy sand), physicochemical parameters, and covered all present land use types (woodland, bushveld, fallow, dry agriculture, and irrigation agriculture) in the Mashare region. In order to compare the ecological similarity of the different soils, 14 physicochemical soil variables (pH, C_*org*_, C/N ratio, conductivity, concentrations of major ions and of nutrients) were determined in March/April 2011 and 2012, in addition to the water content. A Principal Component Analysis (PCA) of these data revealed that the physicochemical soil conditions were distinct for the sandy and the loamy sand soils (PERMANOVA, Df = 1, *F* = 9.6, *p* = 0.001), and among the loamy sands also differed between most land use types (Figure 3A). For most soils, physicochemical parameters changed only little between the two sampling times (Supplementary Figure 1).

Notably, soils with different land use not only differed in their physicochemistry but also showed distinct responses of their water content to the changing precipitation (Figure 2B). The soil water content in woodland and dry agriculture soils decreased from the rainy seasons 2010/2011, 2011/2012 to the weak rainy season 2012/2013 and the dry season 2011, respectively, reflecting the cumulative precipitation of the preceding season. The other soils mostly exhibited a similar trend, however, the loss in water content over the four different sampling campaigns was less consistent. Therefore, the effects of cumulative precipitation, previous water content, land use, and soil type on the soil water content were analyzed by linear modeling over all consecutive sampling intervals (Figure 3B). explaining 39% of the variation (adjusted R^2^). Based on the results, the cumulative precipitation in the preceding season had a slightly positive and statistically significant effect on soil water content. Two types of land use had a strong and significant effect on soil water content. While the effect of irrigation agriculture was anticipated, linear modeling also identified the land use category woodland as an important determinant of soil water content in Mashare soils (Figure 3B).

### Effect of Decreasing Water Availability on the Fraction and Composition of Active Soil Acidobacteria

In the current study, the effects of drought on the active fraction of Acidobacteria were assessed by high-throughput sequencing of cDNA generated from extracted 16S rRNA transcripts. While total cell numbers varied only slightly (Figure 2C), a pronounced significant decrease in the active fraction of Acidobacteria over the 2-year observation period was observed (*p* < 0.01; Figure 2D). Thus, 4.3–12.8% and 1.7–15.1% of the active soil bacterial community were representatives of the acidobacterial phylum in March/April and November 2011, respectively. In March/April 2012 these numbers had decreased to 0.5–5.9%, and subsequently reached the lowest values in March 2013 after the weak rainy season (0.2–2.5%) (Figure 2D and Supplementary Figure 2A). This decline was continuous for all most soil and land use types (Figure 2D). The strongest decline was actually detected after the normal rainy season (November 2011 to March 2012).

Despite the pronounced decrease in abundance values of active Acidobacteria, their composition at the higher taxonomic levels of classes and orders remained rather stable over the four different time points and differed between soil types and land use types (Supplementary Figure 2). Members of the classes *Blastocatellia* and *Vicinamibacteria*, reached highest values in loamy sands, while the “*Acidobacteriia”* dominated in sandy soils (Supplementary Figure 2B). Within the “*Acidobacteriia,”* active representatives of the *Solibacterales* reached higher abundance values than the *Acidobacteriales* and Subgroup 5 in the loamy sands, while *Acidobacteriales* reached higher values in sand soils (Supplementary Figure 2C). Within the class *Blastocatellia*, the *Pyrimonadales* reached a higher fraction of the active community than *Blastocatellales* (Supplementary Figure 2D) while subgroup 6 clearly dominated among the class *Vicinamibacteria* (Supplementary Figure 2E).

**FIGURE 4:**
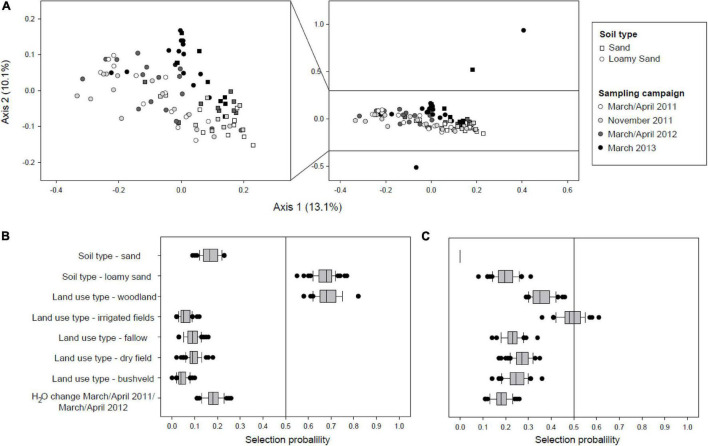
**(A)** β-diversity active acidobacterial communities over 2 year observation period as determined by PCoA of weighted-unifrac distances. For better resolution, the central portion of the ordination plot without three outliers is enlarged on the left. PERMANOVA tests indicated that the changes between the different time points were significant (*p* = 0.001–0.05). **(B)** Variable selection of explanatory environmental variables of Procrustes residuals for the first dry/wet season cycle. **(C)** Variable selection of explanatory environmental variables of Procrustes residuals for the second dry/wet season cycle with severe drought.

In order to detect possible changes in the community composition of active soil Acidobacteria during drought at a higher taxonomic resolution, a multivariate ordination using Principal Coordinate Analysis (PCoA) was performed based on weighted UniFrac distances calculated for acidobacterial sequence variants (Figure 4A). While the community structure was clearly shaped by soil type as indicated by a PERMANOVA test that showed a highly significant effect (*p* = 0.001), a shift in community composition with consecutive sampling times could also be observed. The effect of sampling time was further assessed through pairwise comparisons of the composition of the active Acidobacteria community between the different sampling time points using PERMANOVA. This indicated that the changes between all the different time points were significant (*p* = 0.001–0.05). An additional PERMANOVA analysis containing all four time points demonstrated that sampling time itself had a significant effect (Df = 3, *F* = 3.13, *p* = 0.001).

In order to determine whether and which environmental variables affect changes in the β-diversity of active Acidobacteria, we performed pairwise Procrustes analysis between the three consecutive sampling time points at the end of each rainy season March/April; (see Supplementary Figures 2A,B). Gradient boosting with stability selection was used to identify environmental variables which have high explanatory power on the Procrustes residuals (Lisboa et al., 2014) (response variable). Initial evaluation of the impact of the different numerical soil physicochemical variables showed that the changes in water content between two sampling times had the strongest explanatory power for Procrustes residuals of β-diversity shifts (data not shown). Therefore, the change in soil water content was also included as potential explanatory variable in addition to the categorical variables soil type and land use type. During the first year of the study, the land use type woodland and the soil type loamy sand exerted a measurable impact on the Procrustes residuals (March/April 2011–March/April 2012; Figure 4B) whereas over the second time interval analyzed (April/March 2012 to March 2013) none of the variables tested had sufficient explanatory power to explain the Procrustes residuals and hence the observed changes in β-diversity. While the composition of active Acidobacteria sequence variants clearly shifted over the consecutive seasons with declining rainfalls, the changes of the overall community structure over the first year of the study period could be attributed to responses to certain soil texture and land use rather than soil water content itself.

### Response of Individual Acidobacteria Sequence Variants to Soil Water Availability

**FIGURE 5:**
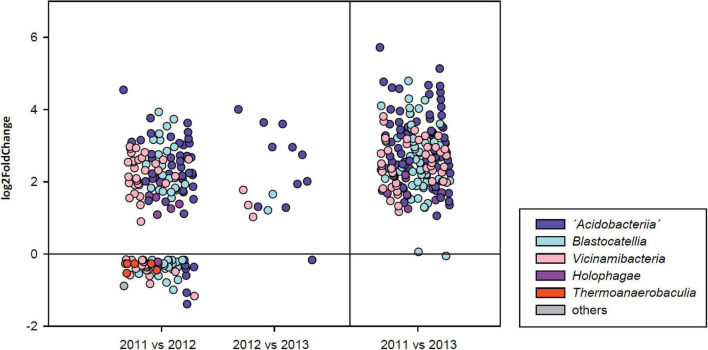
Differential abundance analysis (following ANCOM BC tests) of individual sequence variants after rainy seasons over consecutive 1-year observation periods **(left panel)** and the entire 2-year period **(right panel)**. Different colors reflect the taxonomic affiliation at class level.

A total of 98 sequence variants of active Acidobacteria declined in abundance between March/April 2011 and March/April 2012 samples while only 15 additional SVs were depleted subsequently between March/April 2012 and March 2013. When evaluating the differences between the initial and final sampling dates, a total of 188 active SVs were found to respond to the drought by a decrease in their relative abundance and hence displayed the highest sensitivity to the drought events (Figure 5). Most of these SVs were affiliated with the classes “*Acidobacteriia”* (64), *Blastocatellia* (51) and *Vicinamibacteria* (49). Conversely, a relative enrichment of 58 other SVs was observed over the first year of the observation period. These SVs not only belonged to these three major classes mentioned above, but also comprised several members of the class *Thermoanaerobaculia*. Interestingly, however, a relative enrichment in the second year was only observed for one SVs and only one SV from the family *Pyrinomonadaceae* (class *Blastocatellia*) was found to be enriched in 2013 when compared to samples from 2011, suggesting that this SV, in contrast to all others, is particularly adapted to drought.

To assess whether the observed response of the 188 SVs toward increasing drought displayed a phylogenetic signal and hence was a specific characteristic of certain taxonomic groups, the NRI and NTI values were calculated (Webb et al., 2002). Drought-sensitive SVs were found to be phylogenetically overdispersed (NRI of 0.52, *p* = 0.297) over the entirety of the phylogenetic tree, but some phylogenetic clustering was found toward terminal branches as indicated by a positive NTI of 5.63.

### Specific Responses of Soil Acidobacteria to Continuous or Repeated Drought

**FIGURE 6:**
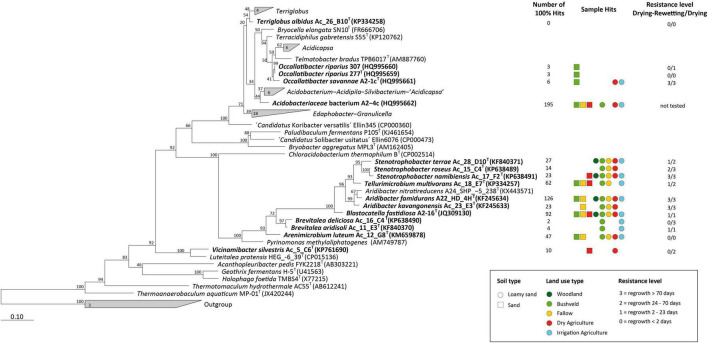
Maximum-likelihood phylogenetic tree (numbers at nodes give percentage of 1,000 bootstrap replicates) of all known acidobacterial isolates with (validly) published names acidobacterial isolates. Numbers in wedges give total numbers of isolates available for the respective genera. The 16 isolates retrieved from Namibian soils are highlighted in bold. Number of 16S rRNA transcripts of these isolates that were detected in sequence datasets from different soils by BLAST (at a 100% identity level), the corresponding land use and soil types, and the type of resistance of the isolates are indicated on the right.

Based on our culture-independent analysis, the effect of drought on the relative abundance of Acidobacteria in Namibian soils was non-linear (Figures 2B,D). The effect on community composition during certain periods was modified by soil type and land use (Figure 4B). For a better understanding of the adaptation of Acidobacteria to changing water content in soils, we conducted controlled laboratory experiments. Over the past years, 15 different strains of Acidobacteria (representing 14 novel species) have been isolated from Namibian soils (Supplementary Tables 2, 3; Foesel et al., 2013, 2015; Huber et al., 2014, 2016; Pascual et al., 2015a,b; Wüst et al., 2016) and validly described. The description of one further Namibian strain—*Acidobacteriaceae* sp. A2-4c -representing another new acidobacterial species will be published soon. The phylogenetic diversity of these 16 available isolates is considerable (Figure 6, marked in bold) and allowed us to assess the drought response for different taxonomic groups from the three Acidobacteria classes that dominate in Namibian soils (“*Acidobacteriia*,” *Blastocatella*, *Vicinamibacteria*; Supplementary Figure 2). The Acidobacteria were inoculated into one standard artificial soil, exposed to drought and their viability was determined.

At the outset, the presence of sequence variants of the 16 isolates in the sequence datasets from the 96 Namibian soils was assessed. The individual 16S rRNA gene sequences of the strains were BLASTed against the dataset of the sequence variants of the Mashare amplicon samples. Interestingly, all strains except *Terriglobus albidus* Ac_26_B10^T^ could be identified with a 100% match in the active fraction of soil Acidobacteria. The number of positively identified hits varied between 2 reads in the case of *Brevitalea deliciosa* Ac_16_C4^T^ and 195 reads in the case of *Acidobacteriaceae* bacterium A2-4c (Figure 6, right). We used 15 acidobacterial strains as models to study the effect of decreasing water availability and heat on viability in long-term laboratory experiments lasting over > 70 days.

**FIGURE 7:**
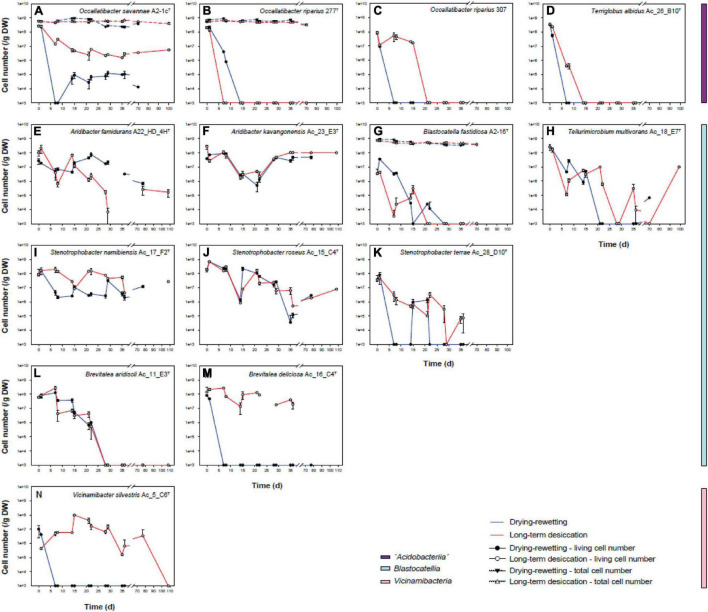
Colony forming units of long-term drying-rewetting (filled circles) and the continuous desiccation experiments (empty circles) with *Occallatibacter savannae* A2-1c^T^ **(A)**, *O. riparius* 277^T^ **(B)** and 307 **(C)**, *Terriglobus albidus* Ac_26_B10^T^ **(D)**, *Aridibacter famidurans* A22_HD_4H^T^ **(E)**, *A. kavangonensis* Ac_23_E3^T^ **(F)**, *Blastocatella fastidiosa* A2-16^T^ **(G)**, *Tellurimicrobium multivorans* Ac_18_E7^T^ **(H)**, *Stenotrophobacter namibiensis* Ac_17_F2^T^ **(I)**, *S. roseus* Ac_15_C4^T^ **(J)**, *S. terrae* Ac_28_D10^T^ **(K)**, *Brevitalea aridisoli* Ac_11_E3^T^ **(L)**, *B. deliciosa* Ac_16_C4^T^ **(M)**, and *Vicinamibacter silvestris* Ac_5_C6^T^ **(N)**.

Individual strains of Acidobacteria were inoculated in sterile artificial soil matrix in two different parallels. The first parallel was incubated at 28°C without any further treatment, resulting in a decline of soil water content over the incubation period from initially 20 to 0%. In the second series of experiments, the effect of repeated drying and rewetting cycles was investigated. For three different strains (*Occallatibacter savannae* A2_1c^T^, *O. riparius* 277^T^ and *Blastocatella fastidiosa* A2-16^T^) we also monitored the total cell numbers during the course of both types of experiments. However, total cell numbers remained stable over the entire incubation period (Figures 7A,B,G).

Distinct changes in the ability to form colonies (viability) were observed for different strains and the two types of desiccation experiments. All strains except *Arenimicrobium luteum* Ac_12_G8^T^ (not shown in Figure 7) survived the treatment and the transfer to the artificial soil matrix. *Occallatibacter savannae* A2-1c^T^ (Figure 7A), *Aridibacter famidurans* A22_HD_4H^T^ (Figure 7E), *A. kavangonensis* Ac_23_E3^T^ (Figure 7F), *Stenotrophobacter namibiensis* Ac_17_F2^T^ (Figure 7I) and *S. roseus* Ac_15_C4^T^ (Figure 7J) survived the repeated water availability conditions and heat treatment in the drying-rewetting experiment quite well and were able to regrow in high numbers at all sampling times until the termination of the experiments. In contrast, *Occallatibacter riparius* 277^T^ (Figure 7B) and 307 (Figure 7C) and *Terriglobus albidus* Ac_26_B10^T^ (Figure 7D) survived only the first drying-rewetting cycle or a short time of continuous desiccation and therefore do not seem to be well adapted to the drought conditions tested. *Blastocatella fastidiosa* A2-16^T^ (Figure 7G), *Tellurimicrobium multivorans* Ac_18_F2^T^ (Figure 7H), *Stenotrophobacter terrae* Ac_28_D10^T^ (Figure 7K) and *Brevitalea aridisoli* Ac_11_E3^T^ (Figure 7L) retained high viability for more than 2 drying-rewetting cycles or several weeks of continuous desiccation, but viability mostly decreased after this period. These four strains showed no regrowth at the later time points 70 and 100 d. Interestingly, two strains, *Brevitalea deliciosa* Ac_16_C4^T^ (Figure 7M) and *Vicinamibacter silvestris* Ac_5_C6^T^ (Figure 7N) did not survive the drying-rewetting experiment even for the first cycle but both strains stayed viable and were capable of forming colonies until day 70 in the parallel long-term desiccation experiment.

## Discussion

### Identification of the Acidobacterial Taxa Responding to Changing Water Availability

To understand the coupling between drought, bacterial diversity, and microbial mediated biogeochemical processes, data on the physiology of desiccation resistance are need for typical representatives of soil bacteria.

In the present study, we assessed the active fraction of Acidobacteria by high-throughput sequencing of cDNA generated from the extracted 16S rRNA transcripts, since in most bacteria, cellular ribosome content is proportional to the growth rate (Vieira et al., 2020). In DNA-based sequence inventories low abundant but highly active and biogeochemically relevant microbial taxa may escape detection and dormant cells and extracellular DNA may confound analysis. In annual grasslands, Acidobacteria were shown to maintain a constant population size (i.e., relative abundance of 16S rRNA genes) whereas their active fraction (based on 16S rRNA transcripts) declined significantly during natural dry-down over several months, and rapidly recovered within hours after rewetting (Barnard et al., 2013). The analysis of 16S rRNA transcripts thus provides a sensitive measure of short-term responses of soil bacterial communities to drought.

### Distinct Effects of Plant Cover and Soil Texture on Acidobacteria During Drought

The percentage of drylands is predicted to increase worldwide over the next decades (Maestre et al., 2015) and especially semiarid regions in the African Southwest likely will be particularly strongly affected (IPCC Climate Change, 2007). In the Kalahari arenosols, a soil type abundant in the latter region, nutrient regeneration seems to be limited by the mineralization potential of soil microorganisms (Wang et al., 2007). Yet, the effects of desiccation on soil microbial communities in semiarid savannah soils have rarely been studied, which makes predictions of future changes in their ecosystem functions difficult. Our results contribute to a better understanding of the response of a major group of soil bacteria to drought.

The effect of aridification on soil bacteria has so far mostly been assessed in field or laboratory desiccation/rewetting experiments (Barnard et al., 2013; Meisner et al., 2018) or through comparison of different soils sampled along aridity gradients (Maestre et al., 2015). The drought that occurred in the years 2012 and 2013 in the Kavango region offered the opportunity to assess its impact systematically in a natural setting and to follow its effects through time in the different soil and land use types. Linear modeling of the determinants of soil water content showed the anticipated effect of direct human intervention through irrigation, but also revealed that woodland cover exerted a strong effect on soil water content during increasing drought (Figure 3). Furthermore, woodland cover, together with the soil matrix, was identified as a driver of the drought-related shifts in the community structure of active Acidobacteria, when variable selection was employed in the analysis of the Procrustes residuals of β-diversity (Figure 4). Together, these results suggest that the plant cover differentially affects the water balance as well as the composition of active soil bacterial communities in the savannah soils. The woodland soils of the Kavango region are characterized by denser vegetation, higher nutrient content, and higher aggregate stability than soils subject to other types of land use, or than more sandy soils (Gröngröft et al., 2013) and therefore may buffer changes in precipitation more effectively.

### Desiccation Response of the Active Acidobacterial Communities in Namibian Soils

In a worldwide analysis across different soils under different aridity regimes, the fraction of Acidobacteria in the total bacterial community declined linearly with aridity (Maestre et al., 2015). Similar observations had been reported for Acidobacteria on rice roots during artificial drought events (Santos-Medellín et al., 2017) where the number of acidobacterial OTUs also directly decreased with decreasing water availability. In contrast, tracking active Acidobacteria over 2 years in the same soils, we observed a steady and significant decrease in the relative fraction of active Acidobacteria despite the considerable intermittent increase of soil water content during the rainy season between November 2011 and March 2012 (Figures 2B,D and Supplementary Figure 3). The physiological response of the entire acidobacterial community to water availability is therefore non-linear, possibly due to different physiological adaptations of the different taxa present. Of note, the diversity of soil acidobacterial communities was previously shown to decline in a non-linear fashion with increasing aridity in contrast to most other bacterial phyla (Maestre et al., 2015).

It has been hypothesized that Acidobacteria in general follow an opportunistic life strategy with declining and increasing ribosomal synthesis during water stress and rewetting, respectively, in contrast to some other phyla like the Actinobacteria (Maestre et al., 2015). This response of Acidobacteria has been suggested to be conserved at the phylum level (Barnard et al., 2013). However, long-term irrigation of a semi-arid alpine pine forest has been shown to not only result in decreased relative abundance of Acidobacteria but also to differentially influence the abundance of acidobacterial taxa on class level (Hartmann et al., 2017). In our study of Kavango soils the relative fractions of active members of the different classes and orders stayed rather constant during the 2-year decline of active Acidobacteria (Supplementary Figures 2B–E). The “*Acidobacteriia”* (mainly the *Acidobacteriales*, *Solibacterales* and subdivision 5, Supplementary Figure 2C) consistently dominated the sandy soils while the classes *Vicinamibacteria* and *Blastocatellia* (*Blastocatellales* and *Pyrimonadales*) always prevailed in the loamy sands (Supplementary Figures 2E,F). These differences in the composition of the acidobacterial classes can be explained by the acidic pH values that prevail in sandy soils and the neutral to basic pH values of loamy sands (Gröngröft et al., 2013). Obviously, members of the three major acidobacterial classes that occur in Kavango dryland soils follow the same patterns with respect to pH that were repeatedly observed in soils of temperate regions (Jones et al., 2009; Naether et al., 2012; Foesel et al., 2014; Ivanova et al., 2020). At first sight, the rather stable ratios of higher Acidobacteria taxa maintained during drought would support the hypothesis of a similar desiccation response across all members of the Acidobacteria, contrary to the conclusions drawn from observations in an alpine soil ecosystems exposed to drought (Hartmann et al., 2017).

However, previous detailed analyses of the acidobacterial community composition across a wide range of different edaphic conditions in temperate grassland soils has provided evidence for a species-specific adaptation of Acidobacteria to other environmental variables including temperature, total nitrogen or phosphorus, or the abundance of protozoa (Foesel et al., 2014). Very recently, systematic niche modeling for 4154 operational taxonomic units of Acidobacteria along gradients of 44 different physicochemical and biological variables revealed a pronounced ecological diversification even at the level of closely related species and recurrent events of convergent evolution that resulted in frequent habitat switching within individual clades of Acidobacteria (Sikorski et al., 2022). The increasing number of representative isolates that has recently become available for the different classes and orders of Acidobacteria now provide the opportunity to also test complex phenotypic traits like resistance to water stress.

### Acidobacterial Strains Respond Differently to Drought

The 15 tested acidobacterial strains exhibited very different capacity for regrowth during the drying-rewetting and the long-term desiccation experiments. Some strains survived for only short time periods of up to 7 days and hence were highly sensitive to drought. Several representatives were able to grow again after the first drying-rewetting cycles or after longer periods of continuous desiccation but were not able form colony forming units after extended exposure. Several strains retained the capability to divide over the entire course of the drying-rewetting and the long-term desiccation experiments and hence were highly resistant to drought (Figure 7).

These different types of desiccation responses observed for laboratory strains of different genera or higher Acidobacteria taxa correspond to the results of our culture-independent analysis of acidobacterial communities (NRI value for SVs depleted over the entire 2 years: 0.52; *p* = 0.297), which indicate that drought sensitivity or persistence does not represent an evolutionarily conserved trait over large phylogenetic distances within the phylum Acidobacteria. For instance, strains which showed the best regrowth capacities were affiliated with the “*Acidobacteriia*” as well as the *Blastocatellia* and therefore were only distantly related. Furthermore, the highly positive NTI value of 5.63 calculated for the drought sensitive SVs suggests that closely related taxa are more likely to be similarly affected by drought (Figure 5). While a similar desiccation response of closely related strains was indeed observed in some instances in our laboratory cultivation experiments (compare *Occalatibacter riparius* strains 277^T^ and 307 (Figures 6, 7B,C), or *Aridibacter famidurans* A22_HD_4H^T^ and *A. kavangonensis* Ac_23_E3^T^; Figures 6, 7E,F), distinct kinetics were observed in several other cases (compare, e.g., *Stenotrophobacter terrae* Ac_28_D10^T^ and *S. roseus* Ac_15_C4^T^, Figures 6, 7J,K). This suggests that a different adaptation to desiccation can even occur on an evolutionary rather short time scale similar to other traits as was shown recently (Sikorski et al., 2022).

### Potential Drought Resistance Strategies of Acidobacteria

So far, little is known on possible strategies of Acidobacteria to withstand desiccation and rewetting. While the formation of exospores or endospores has not been observed and the accumulation of compatible solutes so far has not been studied for the 63 acidobacterial species with (validly) published names, a cellulose synthesis operon that might be involved in capsule formation has been detected in *Acidobacterium capsulatum* DSM 11244^T^ and a class of putatively large proteins detected in “*Solibacter usitatus*” Ellin6076 has been suggested to be potentially involved in desiccation resistance (Ward et al., 2009). Some representatives of the “*Acidobacteriia*” have indeed been reported to produce capsules like “*Acidisarcina polymorpha”* SBC82 (Belova et al., 2018) or EPS like *Granulicella* spp. WH15 and 5B5 (Kielak et al., 2017) which may protect the cells from desiccation. However, this trait has not been described for representatives of the *Blastocatellia* and *Vicinamibacteria* which were shown in the present study to also be able to survive drought for a considerable period of time. To this end, our study has identified isolates that would be suitable targets for future comparative studies of the cellular stress response of different soil Acidobacteria to desiccation.

## Conclusion

In the present study we have shown that the analysis of variances in the acidobacterial activity structure in subtropical soils differing in soil type, land use type and soil water availabilities enables deepened insights in potential control mechanisms of Acidobacteria. The comparison of high-throughput sequences, environmental and physiological data of the several Namibian strains with validly published names enables detailed insights into the interaction patterns of Acidobacteria in subtropical savannah soils of Namibia. Here, we show for the first time that acidobacterial taxa from 96 Namibian soils samples respond differentially to varying soil water contents and proved these findings via physiological tests of isolates from corresponding Namibian soils. Therefore, the isolation and characterization of acidobacterial strains is mandatory for the further understanding of the acidobacterial physiology, their adaptation to the soil environment and their potential contribution to the soil fertility.

## Note

The GenBank/EMBL/DDBJ accession numbers for the 16S rRNA gene sequences of the examined soils are PRJEB46595.

## Data Availability Statement

The datasets presented in this study can be found in online repositories. The names of the repository/repositories and accession number(s) can be found below: https://www.ebi.ac.uk/ena, PRJEB46595.

## Author Contributions

KH and JO wrote the manuscript with support of SV and JS. JO conceived and designed the study. BF designed the drying-rewetting and long-term desiccation experiment which has been performed by KH. KH and PW prepared the 16S rRNA amplicons. AG analyzed the environmental parameters. SV and JS performed the statistics. All authors contributed to the article and approved the submitted version.

## Conflict of Interest

KH, SV, JS, PW, BF, and JO were employed by the non-profit research institution DSMZ. The remaining author declares that the research was conducted in the absence of any commercial or financial relationships that could be construed as a potential conflict of interest.

## Publisher’s Note

All claims expressed in this article are solely those of the authors and do not necessarily represent those of their affiliated organizations, or those of the publisher, the editors and the reviewers. Any product that may be evaluated in this article, or claim that may be made by its manufacturer, is not guaranteed or endorsed by the publisher.

## Footnotes

1 https://www.fao.org/dryland-assessment/en/ (accessed January 12, 2022).

2
https://qiime2.org/

3
https://www.dsmz.de/microorganisms/medium/pdf/DSMZ_Medium1426.pdf
