# Supplementary material for: Differential Response of Acidobacteria to Water Content, Soil Type, and Land Use During an Extended Drought in African Savannah Soils
Source: Front Microbiol. 2022 Feb 11;13:750456. doi: 10.3389/fmicb.2022.750456 (PMC8874233; doi:10.3389/fmicb.2022.750456)
Supplement: Supplementary file 1 [file Data_Sheet_1.pdf]

Differential response of Acidobacteria to water content, soil type, and land use during an extended draught in African savannah soils

Katharina J. Huber[1], Selma Vieira[1], Johannes Sikorski[1], Pia K. Wüst[1], Bärbel U. Fösel[1,2], Alexander Gröngröft[3], and Jörg Overmann[1,4*]

[1] Leibniz Institute DSMZ – German Collection of Microorganisms and Cell Cultures, Braunschweig, Germany

[2] Current address: Research Unit Comparative Microbiome Analysis - Helmholtz Zentrum München – German Research Center for Environmental Health, Biozentrum, Neuherberg, Germany

[3] Institute of Soil Science, Department of Geosciences, University of Hamburg, Hamburg, Germany

[4] Technical University Braunschweig, Braunschweig, Germany

Footnote

The GenBank/EMBL/DDBJ accession numbers for the 16S rRNA gene sequences of the examined soils are PRJEB46595.

* Correspondence: J. Overmann, Leibniz Institute DSMZ – German Collection of Microorganisms and Cell Cultures, Inhoffenstraße 7B, 38124 Braunschweig, Germany. Tel: +49-531-2616-352. Fax: +49-531-2616-418. Email: joerg.overmann@dsmz.de.

Supplementary Tables

Supplementary Table S1: Overview of the 96 soil samples of Mashare/Namibia sampled in March/April 2011 (sampling campaign 1), November 2011 (sampling campaign 2), March/April 2012 (sampling campaign 3) and March 2013, (sampling campaign 4), the corresponding soil types, land use type, water content and total cell number.

Sample	Sampling site	Latitude S	Longitude E	Elevation [m]	Sampling campaign 1/2/3/4	Soil type	Land use type	pH (H_2O)	Water content [%]	Total cell number
**KS-BV-1**	Mashare	17°54´51.91´´	20°12´28.44´´	1087	+/+/+/+	Sand	Bushveld	6.5/6.7/6.2/-	2.60/0.54/0.65/3.55	5.28E+08/4.35E+08/4.89E+08/1.20E+09
**KS-BV-2**	Mashare	17°54´41.26´´	20°10´42.02´´	1069	+/+/+/+	Sand	Bushveld	5.5/6.0/6.7/-	2.31/0.36/2.20/0.87	5.01E+08/2.63E+08/7.09E+08/4.12E+08
**KS-BV-3**	Mashare	17°53´58.85´´	20°12´34.31´´	1085	+/+/+/+	Sand	Bushveld	6.1/6.6/6.8/-	2.80/0.59/4.72/0.34	6.23E+08/3.98E+08/6.80E+08/7.61E+08
**KS-F-1**	Mashare	17°54´21.92´´	20°8´47.98´´	1081	+/+/+/+	Sand	Fallow	5.5/5.4/5.7/-	2.00/0.38/3.42/0.42	1.01E+09/4.52E+08/5.96E+08/3.11E+08
**KS-F-2**	Mashare	17°55´0.16´´	19°15´57.2´´	1084	+/+/+/+	Sand	Fallow	6.2/6.6/6.7/-	5.35/0.49/5.62/0.49	6.70E+08/3.33E+08/3.08E+08/2.28E+08
**KS-F-3**	Mashare	17°55´4.94´´	20°9´6.16´´	1073	+/+/+/+	Sand	Fallow	5.8/6.9/6.8/-	5.18/0.42/0.52/4.22	1.44E+09/4.37E+08/4.08E+08/3.50E+08
**KS-DA-1**	Mashare	17°54´2.63´´	20°13´58.8´´	1079	+/+/+/+	Sand	Dry agriculture	5.3/5.5/5.7/-	2.38/0.93/5.93/0.37	4.38E+08/2.09E+08/6.42E+08/2.15E+08
**KS-DA-2**	Mashare	17°54´9.25´´	20°14´4.52´´	1081	+/+/+/+	Sand	Dry agriculture	8.8/6.5/7.4/-	2.25/0.21/4.59/0.43	4.56E+08/1.76E+08/1.03E+09/3.06E+08
**KS-DA-3**	Mashare	17°54´25.88´´	20°8´49.85´´	1084	+/+/+/+	Sand	Dry agriculture	5.6/5.6/5.7/-	2.23/0.93/5.33/0.82	5.19E+08/3.47E+08/6.75E+08/3.26E+08
**OFP-W-1**	Mashare	17°53´35.52´´	20°14´57.52´´	1061	+/+/+/+	Loamy sand	Woodland	7.9/7.6/7.6/-	13.66/2.18/8.92/4.93	6.37E+09/3.53E+09/3.49E+09/3.28E+09
**OFP-W-2**	Mashare	17°52´37.99´´	20°15´20.84´´	1061	+/+/+/+	Loamy sand	Woodland	7.2/7.1/6.8/-	11.26/1.52/4.68/1.41	1.78E+09/2.74E+09/1.83E+09/6.89E+08
**OFP-BV-1**	Mashare	17°53´34.87´´	20°10´59.2´´	1061	+/+/+/+	Loamy sand	Bushveld	6.8/6.8/6.8/-	5.16/0.89/3.74/1.00	1.58E+09/6.36E+08/2.85E+09/2.67E+09
**OFP-BV-2**	Mashare	17°53´39.26´´	20°13´39.54´´	1051	+/no RNA/+/+	Loamy sand	Bushveld	6.5/7.0/6.6/-	11.63/0.61/4.46/1.07	1.84E+09/6.74E+08/1.22E+09/7.74E+08
**OFP-BV-3**	Mashare	17°53´32.86´´	20°12´25.49´´	1062	+/+/+/+	Loamy sand	Bushveld	6.5/6.7/7.0/-	6.76/0.56/1.41/0.96	1.42E+09/1.01E+09/2.16E+09/1.38E+09
**OFP-BV-4**	Mashare	17°53´33.04´´	20°13´39.79´´	1060	-/only RNA/+/+	Loamy sand	Bushveld	-/-/6.8/-	-/-/4.27/1.09	-/-/4.25E+09/9.47E+08
**OFP-F-1**	Mashare	17°53´42.65´´	20°13´55.16´´	1062	+/+/+/+	Loamy sand	Fallow	6.6/7.3/7.0/-	6.01/0.48/5.78/0.50	6.13E+08/5.50E+08/3.12E+08/2.85E+08
**OFP-F-2**	Mashare	17°53´40.92´´	20°10´39.61´´	1068	+/+/+/+	Loamy sand	Fallow	6.8/7.0/7.0/-	4.38/0.92/3.28/0.57	1.07E+09/5.47E+08/1.36E+09/5.45E+08
**OFP-F-3**	Mashare	17°53´37.93´´	20°14´50.71´´	1069	+/+/+/+	Loamy sand	Fallow	8.2/7.9/7.7/-	7.53/0.43/4.72/0.91	1.24E+09/7.35E+08/1.26E+09/8.61E+08
**OFP-DA-1**	Mashare	17°53´43.66´´	20°13´59.34´´	1063	+/+/+/+	Loamy sand	Dry agriculture	6.9/7.0/7.2/-	4.87/0.66/4.79/0.68	7.30E+08/4.64E+08/1.45E+08/2.24E+08
**OFP-DA-2**	Mashare	17°53´48.84´´	20°9´7.88´´	1061	+/no RNA/+/+	Loamy sand	Dry agriculture	8.4/8.3/8.2/-	9.92/2.70/5.42/1.46	1.85E+09/1.51E+09/7.82E+08/5.55E+08
**OFP-DA-3**	Mashare	17°53´31.78´´	20°10´16.5´´	1069	+/+/+/+	Loamy sand	Dry agriculture	8.2/8.2/8.3/-	7.05/1.69/6.20/1.13	1.42E+09/7.14E+08/6.76E+08/7.45E+08

**OFP-DA-4**	Mashare	17°53´34.4´´	20°13´42.17´´	1060	-/only RNA/+/+	Loamy sand	Dry agriculture	-/-/6.7/-	-/-/4.73/0.65	-/-/9.54E+08/8.83E+08
**OFP-IA-1**	Mashare	17°53´32.14´´	20°10´57.79´´	1066	+/+/+/+	Loamy sand	Irrigated Agriculture	6.1/6.1/5.2/-	9.95/2.90/12.81/6.57	1.10E+09/7.78E+08/1.38E+09/9.67E+08
**OFP-IA-2**	Mashare	17°53´33.86´´	20°12´38.45´´	1062	+/+/+/+	Loamy sand	Irrigated Agriculture	6.7/7.6/7.1/-	10.53/2.16/3.15/2.10	1.27E+09/3.51E+08/1.82E+09/1.33E+09
**OFP-IA-3**	Mashare	17°53´32.39´´	20°11´15.4´´	1064	+/+/+/+	Loamy sand	Irrigated Agriculture	6.6/7.1/6.6/-	14.27/2.71/3.93/3.33	1.02E+09/8.12E+08/1.12E+09/8.76E+08

Supplementary Table S2: Overview of 16 available Acidobacteria isolates from Namibia, their origin and time of sampling (Foesel et. al, 2013; Foesel et. al, 2015; Huber et al., 2014; Huber et al., 2016; Pascual et al., 2015a; Pascual et al., 2015b; Wüst et al., 2016a).

Strain	Soil name/type	Sampling locality	Time point of sampling
Terriglobus albidus Ac_26_B10^{T}	KS-F2	Mashare	March/April 2011
Occallatibacter riparius 277^{T}	rice field	Rundu	March/April 2005
Occallatibacter riparius 307	rice field	Rundu	March/April 2005
Occallatibacter savannae A2-1c^{T}	A2	Erichsfelde	April 2009
Acidobacteriaceae bacterium A2–4c	A2	Erichsfelde	April 2009
Stenotrophobacter terrae Ac_28_D10^{T}	OFP-IA3	Mashare	March/April 2011
Stenotrophobacter roseus Ac_15_C4^{T}	OFP-F3	Mashare	March/April 2012
Stenotrophobacter namibiensis Ac_17_F2^{T}	OFP-W1	Mashare	March/April 2012
Tellurimicrobium multivorans Ac_18_E7^{T}	KS-F2	Mashare	March/April 2011
Aridibacter famidurans A22_HD_4H^{T}	A22	Erichsfelde	April 2009
Aridibacter kavangonensis Ac_23_E3^{T}	OFP-F3	Mashare	March/April 2011
Blastocatella fastidiosa A2-16^{T}	A2	Erichsfelde	April 2009
Brevitalea aridisoli Ac_11_E3^{T}	KS-DA2	Mashare	March/April 2012
Brevitalea deliciosa Ac_16_C4^{T}	OFP-BV2	Mashare	March/April 2011
Arenimicrobium luteum Ac_12_G8^{T}	OFP-IA3	Mashare	March/April 2011
Vicinamibacter silvestris Ac_5_C6^{T}	OFP-W1	Mashare	March 2013

Supplementary Table S3: Overview of two soil sampling sites in Erichsfelde and one soil sampling site in Rundu near the N'Kwazi Lodge (both Namibia) where the strains *Blastocatella fastidiosa* A2-16^{T} (Foesel et. al, 2013), *Acidobacteriaceae* sp. A2-4c, *Occallatibacter savannae* A2-1c^{T} (Foesel et. al, 2015), *Aridibacter famidurans* A22_HD_4H^{T} (Huber et al., 2014), *Occallatibacter riparius* 277^{T} and 307 (Foesel et. al, 2015) where isolated from, respectively. The location of sampling sites is given in Figure 1A.

Sample	Sampling site	Latitude S	Longitude E	Elevation [m]	Sampling time point	Soil type	Land use type	pH (H_2O)
**A2**	Erichsfelde	21°36´41.40´´	16°52´13.40´´	1481	April 2009	Sand	Bushveld	6.11
**A22**	Erichsfelde	21°38´15.80´´	16°52´03.90´´	1497	April 2009	Loamy sand	Bushveld	8.17
**Rice field**	N'Kwazi Lodge (Rundu)	17°51´59.00´´	19°54´24.00´´	1065	March/April 2005	Sand	Rice field	ND

Supplementary Figure S1

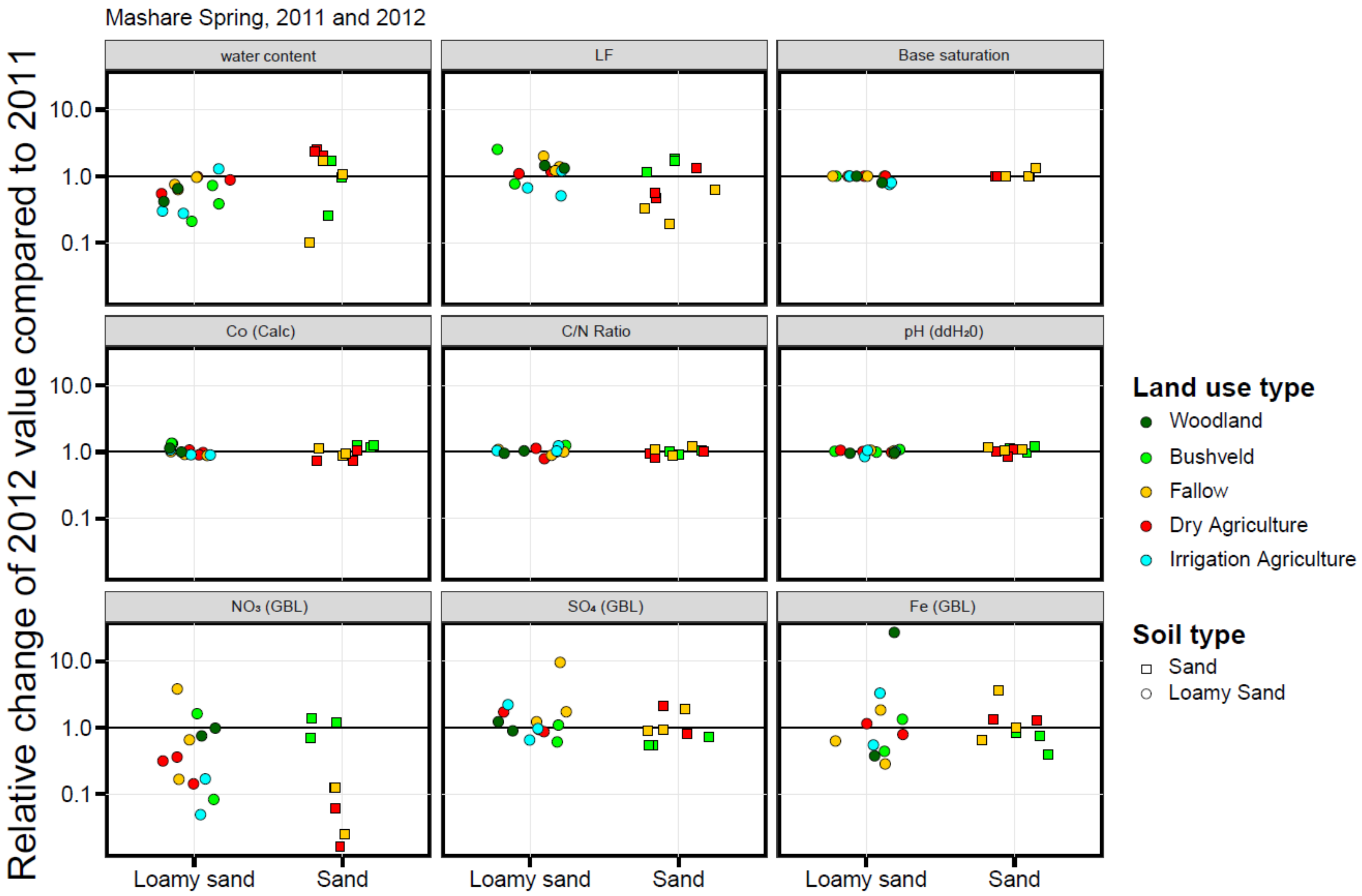

Supplementary Figure S2

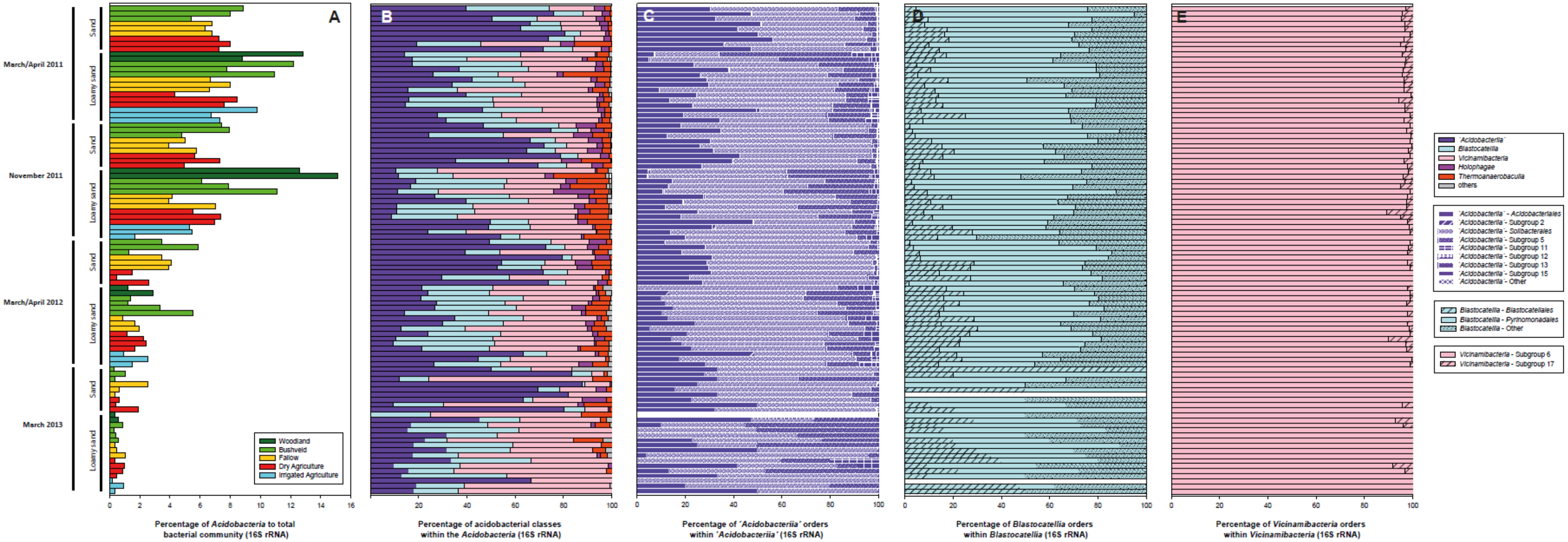

Supplementary Figure S3

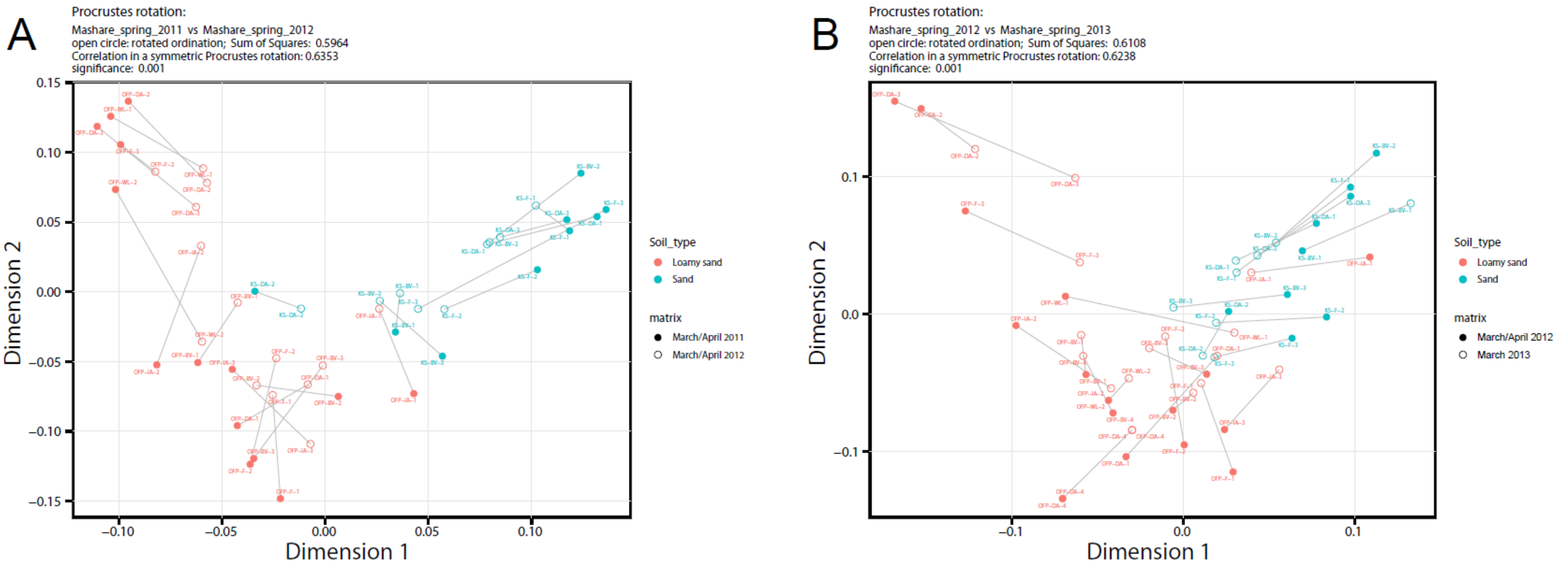

FIGURE LEGENDS

Supplementary Figure S1. Relative change of different environmental parameter values of the sampling campaign March/April 2012 in comparison to the values of the sampling campaign March/April 2011. Values for individual sampling plots are given and denoted according to their soil types (loamy sand, circles; and sand, squares) and land use types (woodland, dark green; bushveld, green; fallow, yellow; dry agricultue, red; irrigation agriculture, blue)**.**

Supplementary Figure S2 A. Percentage of V3 Amplicon-sequences of the phylum Acidobacteria among the V3 Amplicons of Bacteria in the 96 Mashare soil samples. Bars are colored according the land use types woodland (dark green), bushveld (green), fallow (orange), dry agriculture (red) and irrigation agriculture (light blue). **B.** Percentages of members of the acidobacterial classes *´Acidobacteriia´* (purple pale blue), *Blastocatellia* (mint green), *Vicinamibacteria* (rose), *Holophagae* (purple), *Thermoanaerobaculia* (red-orange), and others (grey) within the acidobacterial sequence datasets. **C.** Percentages of the orders *Acidobacteriales* (pale), *Solibacterales* (invers dotted), the subgroups 2 (hatched), 5 (dotted), 11 (grid lines), 12 (diamonds), 13 (arrows), and 15 (white frame) and others (cyclone) within the *´Acidobacteriia´* within the acidobacterial sequence datasets. **D.** Percentages of members of the orders *Blastocatellales* (hatched), *Pyrinomonadales* (pale) and others (dotted) within the *Blastocatellia* within the acidobacterial sequence datasets. **E.** Percentages of the subgroups 6 (pale) and 17 (hatched) within the sequence datasets of the class *Vicinamibacteria*.

Supplementary Figure S3. Procrustes analysis of PCoA plots based on weighted UniFrac distances between soil bacterial communities on **A.** March/April 2011 vs. March/April 2012 and **B.** March/April 2012 vs March/April 2013. Lines connect samples from the same site between the different sampling time points and are proxy for the procrustes residuals.
